# Genetic Variants of EGF and VEGF Predict Prognosis of Patients with Advanced Esophageal Squamous Cell Carcinoma

**DOI:** 10.1371/journal.pone.0100326

**Published:** 2014-06-19

**Authors:** Pei-Wen Yang, Min-Shu Hsieh, Ya-Chuan Huang, Ching-Yueh Hsieh, Tzu-Hsuan Chiang, Jang-Ming Lee

**Affiliations:** 1 Department of Surgery, National Taiwan University Hospital and National Taiwan University College of Medicine, Taipei, Taiwan; 2 Department of Pathology, National Taiwan University Hospital and National Taiwan University College of Medicine, Taipei, Taiwan; The University of Hong Kong, China

## Abstract

**Purpose:**

To investigate the association between genetic polymorphisms of growth factor-related genes and prognosis in patients with advanced esophageal squamous cell carcinoma (ESCC).

**Patients and Methods:**

A total of 334 ESCC patients with advanced tumor stages (stages IIB, III and IV) were enrolled in the study. The genotypes of 14 candidate single nucleotide polymorphisms (SNPs) involved in growth factor-related functions were analyzed using iPLEX Gold technology from the genomic DNA of peripheral leukocytes, and were correlated with the clinical outcome of patients. Serum levels of growth factors were examined by enzyme-linked immunosorbent assay (ELISA).

**Results:**

The genetic polymorphisms of EGF:rs4444903, EGF:rs2237051 and VEGF:rs2010963 showed significant associations with overall survival (OS) of advanced ESCC patients (A/A+ A/G vs. GG, [HR = 0.77, 95% CI = 0.60–0.99, P = 0.039 for rs4444903; A/G+ G/G vs. A/A, [HR = 0.74, 95% CI = 0.58–0.95, P = 0.019 for rs2237051; G/G+G/C vs. C/C, [HR] inves = 0.69, 95% CI = 0.50–0.95, P = 0.023 for rs2010963). EGFR:rs2227983 and 3 SNPs of PIK3CA also showed borderline significant correlation with OS of advanced ESCC patients (P = 0.058 for rs2227983; P = 0.069, 0.091 and 0.067 for rs6443624, rs7651265 and rs7621329 of PIK3CA respectively). According to cumulative effect analysis of multiple SNPs, patients carrying 4 unfavorable genotypes exhibited more than a 3-fold increased risk of mortality. Finally, both EGF and VEGF expression levels significantly associated with patient mortality.

**Conclusion:**

The genetic variants and expression levels of EGF and VEGF can serve as prognostic predictors in patients with advanced ESCC, and thus provide more information for optimizing personalized therapies for patients with ESCC.

## Introduction

Esophageal cancer is a deadly disease worldwide [1], [2]. The prognosis of esophageal cancer is relatively poor. Under multimodality therapy combining chemoradiation and surgery, a 2-year survival rate ranges from only around 30 to 68% and is highly dependent on tumor stage [3], [4], [5]. The 2-year survival rate of patients with stage III or IV tumors has been reported to be only around 30–46% [3], [5]. In histology, esophageal cancer presents mainly as either esophageal squamous cell carcinoma (ESCC) or esophageal adenocarcinoma (EA) [1]. EA is closely associated with Barrett’s esophagus and represents the most common cell type of esophageal cancer in the Caucasian population [6]. ESCC is more prevalent in the non-Caucasian populations and more correlated with environmental factors, such as smoking, alcohol consumption and betelnut chewing [7], [8].

Growth factors are usually proteins or steroid hormones and act as signaling molecules that regulate various cellular responses such as cell proliferation, survival and differentiation. Many traditional growth factors stimulate cellular response by binding to specific receptors which associate with tyrosine kinase activity [9]. Interactions between growth factor ligands and receptor tyrosine kinase lead to activation of tyrosine kinase activities and activate signaling pathways via downstream molecules containing Src homology 2 (SH2) domains [9], [10]. Since growth factor–mediated signaling pathways promote the events of cell growth, over-activation of the signaling pathways usually highly correlates with the transformation process in various types of cancer. Thus, evaluation of their potential role as predictive biomarkers has become an exciting approach used in the treatment of cancer.

Epidermal growth factor receptor belongs to the ErbB family of tyrosine kinase receptors, which includes EGFR (ErbB-1), HER-2/*neu* (ErbB-2), HER-3 (ErbB-3), and HER-4 (ErbB-4), all known to be involved in modulating pathways of tumor growth or proliferation [11]. Genetic variants of EGFR have also been shown to influence clinical outcome of many different types of cancer including non-small-cell lung cancer (NSCLC) [12], [13], prostate cancer [14], metastatic colorectal cancer (mCRC) [15], and pancreatic cancer [16]. Most studies have focused on the R497K (rs2227983) and the CA repeat polymorphisms within intron 1, as EGFR R497K is known to attenuate the binding of the ligand EGF while the CA repeat polymorphism appears to significantly influence the transcription of *EGFR* [17]. The interaction of EGFR R497K with EGF at the +61A/G (rs4444903) polymorphism has been shown to enhance the risk of esophageal cancer [18]. We also found that the polymorphism in EGFR intron 1 was able to predict the prognosis of our patients with esophageal cancer after chemoradiation and surgery previously [19].

Vascular endothelial growth factor (VEGF) is the most potent endothelial growth factor. VEGF stimulates the growth of new blood vessels, regulates vascular permeability and provides an anti-apoptotic effect in endothelial cells [20]. It is frequently over-expressed in patients with esophageal cancers and has been suggested to contribute to tumor progression by stimulating angiogenesis [21] and to associate with poor clinical outcome in patients with more advanced esophageal squamous cell carcinoma [22]. The association of the SNPs of VEGF with clinical outcome of patients with esophageal cancer has been clarified previously [23].

Insulin and Insulin-like growth factor-1 (IGF-1) are able to regulate cell growth by binding to insulin receptor or insulin-like growth factor-I receptor (IGF-IR). Growing evidence supports the roles of insulin and IGF-1 as vital growth factors which play an essential role in the proliferation of tumor cells [24]. The genetic polymorphisms of the insulin-related pathway are associated with the risk of colorectal cancer [25] and prostate cancer [26]. However, the relationship between insulin-related SNPs and esophageal cancer remains mostly unknown.

The PI3K/PTEN/AKT/mTOR pathway is the one of the major downstream pathways that responds to growth factor-mediated stimulation. The SNPs of the genes involved in the PI3K/PTEN/AKT/mTOR pathway have recently been demonstrated to be associated with treatment outcome in esophageal cancer patients who have undergone surgery and chemoradiotherapy [27]. The patients enrolled in the aforementioned study included 174 cases with adenocarcinoma and 36 cases with squamous cell carcinoma. Two SNPs within the genes AKT2 and FRAP1 (encoding mTOR) were correlated with poor treatment response, while a better response was associated with heterozygosity for AKT1 rs3803304.

Growing reports revealed the significant prognostic relevance of the SNPs among the genes involved in growth factor-mediated pathways in cancers. In current study, we systematically investigated the prognostic effects of growth factor-related SNPs in ESCC patients with advance-stage in terms of their impacts on survival and tumor recurrence.

## Materials and Methods

### Study Population

The study was analyzed retrospectively. The subjects consisted of 334 ESCC patients treated in the surgical department of National Taiwan University Hospital from 1996 to 2011. The inclusion criteria included both male and female patients histologically confirmed with stage IIb, III or IV primary esophageal squamous cell carcinoma according to the AJCC staging system [28]. The exclusion criteria included pregnant women, pediatric patients, and those unable to give informed consent. The participants in the study provide their written informed consent. The study and consent procedure have been approved by the Research Ethics Committee of National Taiwan University Hospital (201012101RC). Information regarding demographics, tumor location, TNM stage (according to the AJCC 7^th^ edition) [28], course of treatment, and vital and recurrence status was obtained from reports kept at the Tumor Registry of National Taiwan University and/or from medical chart-review.

Each patient’s buffy coat was isolated from 10 ml of blood obtained before treatment and stored in a −80°C freezer for DNA extraction. Cisplatin-based neoadjuvant concurrent chemoradiation therapy (CCRT) was administered to patients with locally advanced disease (presence of T3 or N1 disease or more advanced), diagnosed by endoscopic ultrasound or computed tomography, and consisted of cisplatin combined either with 5-FU or paclitaxel and concomitant 4000 cGy of radiation. Esophagectomy and esophageal reconstruction with gastric or colonic interposition was performed on those patients with resectable disease status and acceptable surgical risk after CCRT. Definitive CCRT with 6000 cGy of radiation or best supportive care was given to those patients deemed unsuitable or unwilling to undergo surgical resection. Overall survival (OS) duration was defined as the interval between initial diagnosis or surgical resection of the disease and mortality of the patient, whereas progression-free survival (PFS) was defined as the interval between diagnosis or surgical resection of the disease and detection of local recurrence and disease progression of the tumor or death. The patients were enrolled from 1996 and followed up until June 2011. The rate of patient loss to follow-up was 9.9% (33/334), and the median follow-up time was 81 months [29].

### DNA Extraction

The buffy coat was isolated from a 10 ml whole blood sample obtained from each patient before treatment. Genomic DNA was extracted from the buffy coat containing the PBMCs (peripheral blood mononuclear cells) using the QIAamp DNA mini kit (Qiagen, Hamburg Germany) following manufacturer’s instructions. The DNA was dissolved in TE buffer and stored in a −20°C freezer.

### SNP Genotyping

We selected the candidate SNPs (Table S1) mostly based on previous studies investigating their association with cancer risk or prognosis. The candidate SNPs at *EGFR*, *EGF* [30], [31], VEGF [32] and the factors involved in the PI3K/PTEN/AKT/mTOR pathway [33] were selected mostly based on reports analyzing esophageal cancer risk or prognosis. The candidate SNPs at *IGF1* and *IGF1R* were selected based on research of metastatic CRC patients [34]. Genotypings were determined using MassARRAY iPLEX Gold technology according to the manufacturer’s instructions (San Diego, USA). PCR-primers and extension-primers were analyzed and designed by using SeqTool Document v1.0 (IBMS, Taiwan). The results of genotyping were manually confirmed using MassARRAY TyperAnalyzer v3.3 software (Sequenom).

### Enzyme-Linked Immunosorbent Assay (ELISA)

A total of 115 advanced-stage ESCC subjects were randomly selected for analysis of EGF and VEGF expression. Both EGF and VEGF expression levels in the sera of ESCC patients were determined by ELISA (enzyme-linked immunosorbent assay) using human EGF and VEGF (VEGF-A, VEGF-165) ELISA kits (Invitrogen, Camarillo, CA) according to the manufacturer’s instructions. For each reaction, one hundred µl of serum were used for EGF detection and 50 µl for VEGF detection. Reactions were performed in duplicate.

### Statistical Analysis

Pearson’s χ^2^ test or Fisher’s exact test was used as appropriate to investigate the association between individual genetic polymorphisms and clinical outcome of the cohort. Multivariate Cox proportional hazards regression was used to evaluate the adjusted Hazard radios (HRs) of death and disease progression including all the potential significant covariates for analysis. Data were expressed as mean value and 95% confidence interval (CI). The crude correlation between genotypes and survival were estimated using the Kaplan-Meier method and assessed using the log-rank test. The association between EGF or VEGF expression and patient genotype were analyzed using a t-test and displayed by box-plot. The effect of EGF or VEGF expression on the survival of patients was also analyzed by multivariate Cox proportional hazards regression and the Kaplan-Meier method. All statistical analyses were conducted with SPSS, version17.0 (SPSS Institute, Chicago, IL). A two-sided *P*-value less than 0.1 (in stage I analysis) or 0.05 (in stage II analysis) was considered statistically significant.

## Results

A total of 334 ESCC patients with advanced tumor stages (stage IIB, III and IV) were enrolled in the study. Among these subjects, 309 patients (92.51%) were men, 268 patients (80.24%) were diagnosed with stage III or IV, 144 patients (43.11%) received esophagectomy, and 246 patients (73.65%) were treated with CCRT (concurrent chemoradiotherapy) (Table 1). Tumor stage and treatment with esophagectomy (or not) were significantly associated with risk of death and recurrence (P<0.001 in death and P = 0.001 in recurrence for stages; P = 0.040 in death and P = 0.013 in recurrence for esophagectomy; Table 1). Both age and treatment with CCRT (or not) showed borderline significant correlation with survival (P = 0.055 for CCRT; P = 0.070 for age; Table 1).

**Table 1:** Prognostic effects of demographic and clinical factors on survival and recurrence in ESCC patients with advanced tumor stages.

Variable	Dead	Alive	p-value	Recurrence	No recurrence	p-value
	N = 274	N = 60		N = 299	N = 35	
**Stage**						
IIB	43 (65.2)	23 (34.8)	**<0.001**	52 (78.8)	14 (21.2)	**0.001**
III and IV	231 (86.2)	37 (13.8)		247 (92.2)	21 (7.8)	
**Esophagectomy**						
Yes	111 (77.1)	33 (22.9)	**0.040**	122 (84.7)	22 (15.3)	**0.013**
No	163 (85.8)	27 (14.2)		177 (93.2)	13 (6.8)	
**CCRT**						
No	57 (85.1)	10 (14.9)	0.055	59 (88.1)	8 (11.9)	0.270
Yes	196 (79.7)	50 (20.3)		219 (89.0)	27 (11.0)	
CT+RT	21 (100)	0 (0)		21 (100)	0 (0)	
**Gender**						
Male	255 (82.5)	54 (17.5)	0.414	278 (90.0)	31 (10.0)	0.494
Female	19 (76.0)	6 (24.0)		21 (84.0)	4 (16.0)	
**Age**						
<50	60 (77.9)	17 (22.1)	0.070	69 (89.6)	8 (10.4)	0.695
50–65	112 (78.9)	30 (21.1)		125 (88.0)	17 (12.0)	
>65	102 (88.7)	13 (11.3)		105 (91.3)	10 (8.7)	

To select potential prognostically relevant SNPs, we randomly selected 95 subjects to conduct a pre-test by analyzing 26 growth-factor related SNPs, including 8 SNPs in *EGFR*; 2 SNPs in *EGF*; 2 SNPs in *IGF1R*; 2 SNPs in *IGF*; 4 SNPs in *VEGF*; 3 SNPs in *PIK3CA*; and a single SNP in *AKT1*, *AKT2*, *FRAP1* and *PTEN* (Table S1). The prognostic effects of these SNPs were analyzed using multivariate Cox regression analysis. Fourteen SNPs displayed significant or borderline significant association with overall survival (Table S1). The prognostic relevance of these growth-factor related SNPs was also investigated in 80 ESCC patients with earlier stages (Stage I or IIa). No SNPs displayed association with the survival of these patients (data not shown).

Combining 239 independent subjects, these 14 candidate SNP were further analyzed in all subjects (N = 334, Table 2). Both SNPs in EGF, rs4444903 and rs2237051, exhibited significance for overall survival (Table 2). Patients carrying major allele A in EGF:rs4444903 exhibited obvious reduced risk for death ([HR] = 0.77, 95% CI = 0.60–0.99, P = 0.039; Table 2), whereas patients with the minor allele G in EGF:rs2237051 showed significantly better survival ([HR] = 0.74, 95% CI = 0.58–0.95, P = 0.019, Table 2). The wildtype genotype of VEGF:rs2010963 was also significantly associated with improved survival ([HR] = 0.69, 95% CI = 0.50–0.95, P = 0.023; Table 2). EGFR:rs2227983 and 3 SNPs in *PIK3CA* also exhibited borderline significant correlation with survival (P = 0.058 for EGFR:rs2227983; P = 0.069 for PIK3CA:rs6443624; P = 0.091 for PIK3CA:rs7651265; P = 0.067 for PIK3CA:rs7621329; Table 2). Even though none of these genetic polymorphisms significantly predicted tumor recurrence among these patients, EGFR:rs2227983, VEGF:rs2010963, and EGF:rs2237051 showed borderline significance for disease progression (P = 0.058 for EGFR:rs2227983, P = 0.073 for VEGF:rs2010963, and P = 0.104 for EGF:rs2237051; Table 2).

**Table 2:** The effect on overall and progression-free survival of the candidate SNPs in patients with advanced ESCC under multivariate analysis.

		ESCC patients with advanced tumor (N = 334)						
					Overallsurvival		Progression-freesurvival	
Gene/SNPs	Functiontypes	Nucleotidechanges	Model	MAF	*HRs(95% CI)	p-value	*HRs(95% CI)	p-value
**EGFR**								
rs2227983	Missense	G/A	ADD	28.4%	1.17 (1.00−1.37)	0.058	1.16 (1.00−1.34)	0.058
**EGF**								
rs4444903	5′UTR	A/G	REC	48.5%	0.77 (0.60−0.99)	**0.039**	0.88 (0.70−1.11)	0.285
rs2237051	Missense	A/G	DOM	9.3%	0.74 (0.58−0.95)	**0.019**	0.82 (0.65−1.04)	0.104
**VEGF**								
rs2010963	5′UTR	G/C	REC	16.8%	0.69 (0.50−0.95)	**0.023**	0.75 (0.55−1.03)	0.073
rs3025039	5′UTR	C/T	ADD	4.5%	0.99 (0.80−1.23)	0.946	0.97 (0.80−1.19)	0.773
**IGF1R**								
rs2272037	Intron	G/A	ADD	8.1%	1.10 (0.92−1.32)	0.294	1.06 (0.89−1.27)	0.502
rs2016347	3′UTR	A/C	ADD	22.8%	1.09 (0.91−1.30)	0.358	1.03 (0.87−1.23)	0.727
**IGF1**								
rs7136446	Intron	T/C	ADD	3.3%	1.09 (0.86−1.36)	0.485	1.03 (0.82−1.28)	0.827
**PIK3CA**								
rs6443624	Intron	C/A	DOM	1.5%	1.33 (0.98−1.80)	0.069	1.21 (0.90−1.62)	0.216
rs7651265	Intron	A/G	DOM	1.5%	1.31 (0.96−1.78)	0.091	1.17 (0.86−1.58)	0.312
rs7621329	Intron	C/T	DOM	1.5%	1.33 (0.98−1.81)	0.067	1.18 (0.88−1.60)	0.267
**AKT1**								
rs1130214	5′UTR	G/T	ADD	2.1%	0.90 (0.69−1.17)	0.425	0.86 (0.66−1.11)	0.235
**AKT2**								
#rs892119	Intron	G/A	REC	24.3%	0.83 (0.62−1.10)	0.194	0.86 (0.65−1.13)	0.270
**FRAP1**								
rs11121704	Intron	T/C	ADD	0%	1.22 (0.83−1.80)	0.315	1.13 (0.78−1.64)	0.525

*Adjusted for age, gender, stage, op and CCRT status.

#with 2 missing values.

ADD: additive; DOM: dominant; REC, recessive;

MAF: minor allele frequency.

We summarized the prognostically favorable and unfavorable genotypes of these prognostically relevant SNPs in Table 3. A joint analysis of the cumulative effects of the unfavorable genotypes on survival was further conducted. Patients with both unfavorable genotypes of EGF:rs4444903 and EGF:rs2237051 exhibited a 1.41-fold increased risk of mortality compared to patients without either of these unfavorable genotypes ([HR] = 1.41, 95% CI = 1.08–1.85, P = 0.013, P trend = 0.015; Table 4). These two EGF SNPs separately analyzed in combination with VEGF:rs2010963 resulted in more significant joint effects. Patients with both unfavorable genotypes of VEGF:rs2010963 and either EGF:rs4444903 or EGF:rs2237051 had an approximately 1.7-fold increased risk of death ([HR] = 1.70, 95% CI = 1.06–2.72, P = 0.027, P trend = 0.003 for EGF:rs4444903 with VEGF:rs2010963; [HR] = 1.74, 95% CI = 1.09–2.80, P = 0.022, P trend = 0.002 for EGF:rs2237051 with VEGF:rs2010963; Table 4). The cumulative effect of VEGF:rs2010963 with either EGF:rs4444903 or EGF:rs2010963 is better than the effect of all the 3 SNPs combined (P = 0.075). We further jointly analyzed EGFR:rs2227983 and PIK3CA:rs6443624. The hazard for death further increased to 3.29 or 3.14-fold in patients carrying the four unfavorable genotypes of VEGF, EGFR, PIK3CA and either EGF:rs2237051 or EGF:rs4444903 ([HR] = 3.29, 95% CI = 1.36–7.96, P = 0.008 and [HR] = 3.14, 95% CI = 1.12–8.19, P = 0.008, respectively; Table 4). A strong significant trend of increasing risk of death or recurrence with increasing numbers of unfavorable genotypes was noted in both combined analyses (P trend<0.001 for OS and P = 0.004 for PFS in EGF:rs2237051 set; P<0.001 for OS and P = 0.011 for PFS in EGF:rs4444903 set; Table 4).

**Table 3:** Favorable and unfavorable genotypes of the growth factor-related genes for the prognosis of patients with advanced ESCC.

SNPs	Favorable genotype(s)	Unfavorable genotype(s)
EGF:rs4444903	A/A, A/G	G/G
EGF:rs2237051	A/G, G/G	A/A
VEGF:rs2010963	G/G, G/C	C/C
EGFR:rs2227983	G/G	G/A, A/A
PIK3CA:rs6443624	C/C	C/A, A/A
PIK3CA:rs7651265	A/A	A/G, G/G
PIK3CA:rs7621329	C/C	C/T, T/T

**Table 4:** Cumulative effects of the unfavorable genotypes on the prognosis of patients with advanced ESCC.

		ESCC patients withadvanced tumor (N = 334)			
		Overallsurvival		Progression-freesurvival	
No. of unfavorablegenotypes	N	Adjusted HRs(95% CI)	p-value	Adjusted HRs(95% CI)	p-value
**EGF:rs4444903+EGF:rs2237051**					
0	155	1		1	
1	45	0.95 (0.65−1.39)	0.795	0.77 (0.53−1.18)	0.169
2	134	1.41 (1.08−1.85)	**0.013**	1.23 (0.96−1.58)	0.107
Trend		1.18 (1.03−1.36)	**0.015**	1.10 (0.97−1.25)	0.140
**EGF:rs4444903+VEGF:rs2010963**					
0	144	1		1	
1	162	1.40 (1.08−1.80)	**0.011**	1.22 (0.96−1.55)	0.107
2	28	1.70 (1.06–2.72)	**0.027**	1.37 (0.88−2.13)	0.164
Trend		1.34 (1.10−1.63)	**0.003**	1.19 (0.99−1.43)	0.063
**EGF:rs2237051+VEGF:rs2010963**					
0	154	1		1	
1	153	1.43 (1.11−1.85)	**0.006**	1.32 (1.03–1.68)	0.027
2	27	1.74 (1.09−2.80)	**0.022**	1.40 (0.89−2.18)	0.144
Trend		1.37 (1.13−1.66)	**0.002**	1.24 (1.03−1.48)	**0.022**
**EGF:rs4444903+EGF:rs2237051** **+VEGF:rs2010963**					
0	129	1		1	
1	66	1.08 (0.77−1.51)	0.654	0.91 (0.66−1.26)	0.562
2	114	1.55 (1.16−2.08)	**0.003**	1.34 (1.02−1.76)	0.034
3	25	1.58 (0.96−2.61)	0.075	1.25 (0.78−2.00)	0.352
Trend		1.21 (1.07−1.37)	**0.002**	1.10 (0.97−1.25)	0.140
**EGF:rs2237051+VEGF:rs2010963** **+EGFR:rs2227983**					
0	51	1		1	
1	147	1.24 (0.86−7.79)	0.247	1.26 (0.89−1.78)	0.195
2	118	1.74 (1.19−2.53)	**0.004**	1.66 (1.16−2.37)	0.005
3	18	2.37 (1.25−4.50)	**0.008**	1.52 (0.83−2.76)	0.172
Trend		1.34 (1.14−1.57)	**<0.001**	1.23 (1.07−1.42)	**0.005**
**EGF:rs2237051+VEGF:rs2010963** **+EGFR:rs2227983** **+PIK3CA:rs6443624**					
0	45	1		1	
1	132	1.37 (0.93−2.03)	0.115	1.35 (0.93−1.97)	0.114
2	113	1.76 (1.18−2.63)	**0.006**	1.80 (1.23–2.63)	**0.002**
3	37	2.11 (1.27−3.50)	**0.004**	1.63 (1.02−2.61)	**0.042**
4	7	3.29 (1.36−7.96)	**0.008**	1.68 (0.70−4.05)	0.247
Trend		1.30 (1.14−1.48)	**<0.001**	1.19 (1.06−1.34)	**0.004**
**EGF:rs4444903+VEGF:rs2010963** **+EGFR:rs2227983**					
0	47	1		1	
1	144	1.53 (1.05–2.21)	**0.026**	1.46 (1.03−2.09)	**0.036**
2	125	1.78 (1.22−2.60)	**0.003**	1.62 (1.13−2.32)	**0.009**
3	18	2.41 (1.26−4.59)	**0.008**	1.64 (0.89−3.00)	0.112
Trend		1.30 (1.11–1.51)	**0.001**	1.19 (1.04−1.38)	**0.015**
**EGF:rs4444903+VEGF:rs2010963** **+EGFR:rs2227983** **+PIK3CA:rs6443624**					
0	42	1		1	
1	126	1.61 (1.08−2.40)	**0.020**	1.49 (1.02−2.18)	**0.039**
2	122	1.94 (1.30−2.89)	**0.001**	1.87 (1.28−2.73)	**0.001**
3	38	2.08 (1.24–3.47)	**0.005**	1.59 (0.98−2.57)	0.059
4	6	3.14 (1.20−8.19)	**0.020**	1.57 (0.60−4.07)	0.358
Trend		1.27 (1.12−1.45)	**<0.001**	1.17 (1.04−1.31)	**0.011**

**Figure 1:**
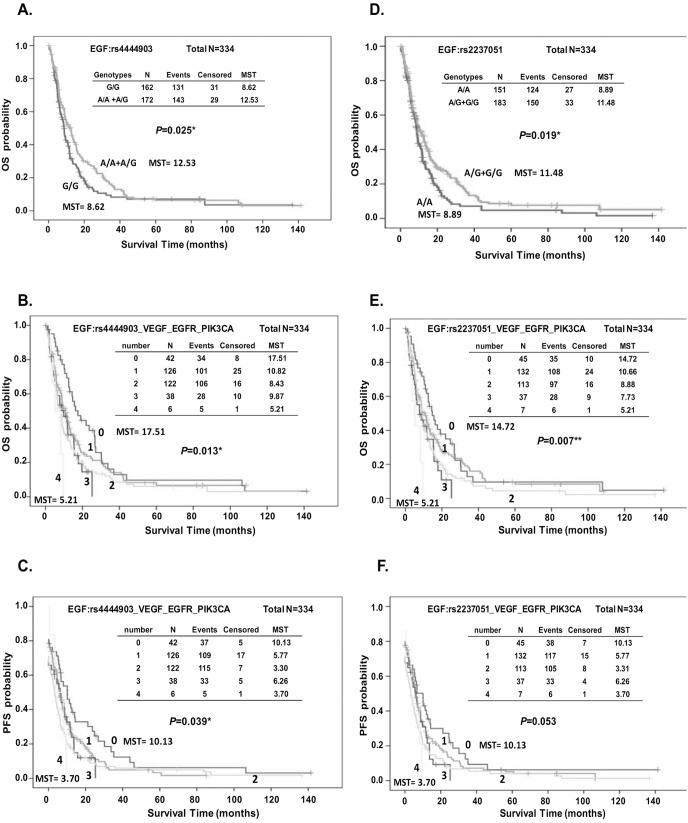
Kaplan-Meier estimates of overall survival (OS) (A, B, D, and E) or PFS (C, F) by genotype of EGF:rs4444903 (A) and EGF:rs2237051 (D) alone, or in combination with unfavorable genotypes of VEGF, EGFR and PIK3CA (B, C, E, and F).

The Kaplan-Meier survival function for OS and PFS in all patients grouped by the genotypes of EGF: rs4444903 and EGF:rs2237051 was analyzed. Compared with those carrying favorable genotypes, patients with the adverse genotypes had a significantly reduced survival duration (MST = 12.53 vs. 8.62 months, log-rank P = 0.025 for EGF:rs4444903, Fig. 1A; MST = 12.53 vs. 8.62 months, log-rank P = 0.019 for EGF:rs2237051, Fig. 1B). Overall survival also differed significantly among the group of advanced ESCC patients according to the accumulated number of unfavorable genotypes within EGF, VEGF, EGFR and PIK3CA (log-rank P = 0.013 in EGF:rs4444903-containing set, and log-rank P = 0.007 in EGF:rs2237051-containing set, Fig. 1C and 1D respectively). Patients carrying all of the unfavorable genotypes exhibited significantly increased risk of death compared with those carrying none of the adverse genotypes (MST = 17.51 vs. 5.21 months in EGF:rs4444903-containing set, and MST = 14.72 vs. 5.21 months in EGF:rs2237051-containing set, Fig. 1C and 1D respectively). The progression-free survival was also significantly different among patients grouped by the number of unfavorable genotypes carried in the EGF:rs4444903-containing set (log-rank, P = 0.039 Fig. 1E) and borderline significantly different in the EGF:rs2237051-containing set (log-rank P = 0.053, Fig. 1F).

**Figure 2:**
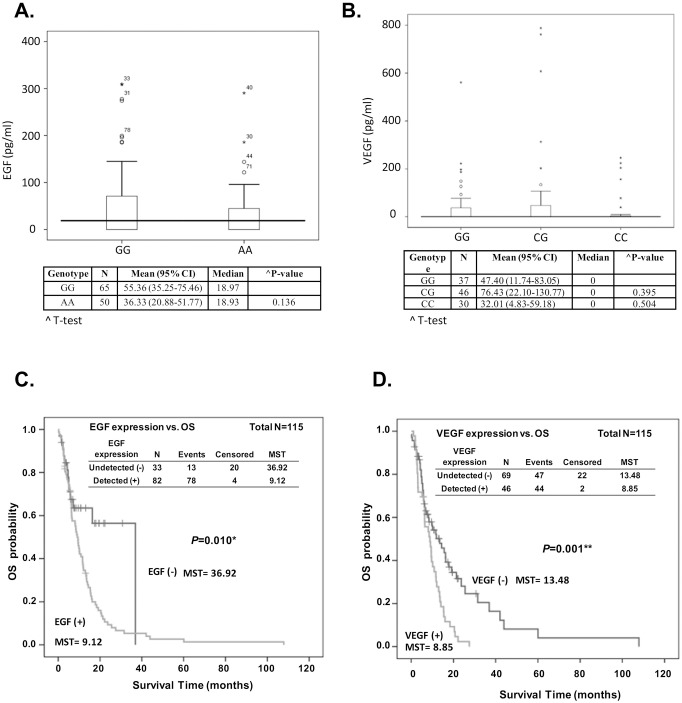
The box-plot distributions of serum EGF (A) and VEGF (B) levels of 115 advanced ESCC patients are displayed by genotype of rs4444903 (A) and rs2010963 (B) respectively. Kaplan-Meier estimates of overall survival (OS) by detected or undetected expression of EGF (C) or VEGF (D).

The rs4444903 SNP, localized at position 61 in the 5′-untranslated region (5′UTR) of EGF, has been found to be correlated with production level of EGF measured in peripheral-blood mononuclear cells *in-vitro* [35]. Meanwhile, rs2010963 at the 5′-UTR of VEGF has also been suggested to modulate VEGF level [36]. We then investigated whether the genetic polymorphisms within the 5′UTR were associated with expression levels of EGF or VEGF. We randomly selected 115 subjects from these patients. The levels of EGF and VEGF in the serum of each patient were determined by ELISA. As expected, homozygous GG carriers of rs4444903 showed a trend of increased EGF level compared with AA carriers (Fig. 2A, T-test P = 0.136). However, we did not find an association between the genotypes of rs2010963 and serum VEGF level (Fig. 2B, T-test P = 0.395 for CG vs. GG and P = 0.504 for CC vs. GG). We further observed the impact of EGF and VEGF on the clinical outcome of patients. Kaplan-Meier survival analysis revealed that the group with detectable levels of EGF had a significantly increased risk of death compared to the group with undetectable levels (MST = 36.92 vs. 9.12 months, log-rank P = 0.010, Fig. 2C). A similar result was observed in the VEGF analysis. Patients with detectable VEGF showed an increased risk for poor prognosis (MST = 13.48 vs. 8.85 months, log-rank P = 0.001, Fig. 2D). Finally, the logistic regression model also demonstrated that patients with detectable VEGF exhibited a higher risk of death as well as recurrence ([OR] = 1.89, 95% CI = 1.22–2.92, P = 0.005 for death; [OR] = 1.57, 95% CI = 1.04–2.38, P = 0.032 for recurrence; see Table S2).

## Discussion

A growing body of research shows a significant association between SNPs of the genes involved in growth factor mediated pathways and prognosis of esophageal adenocarcinoma cancer. We here have demonstrated that the genetic polymorphisms rs4444903 and rs2237051 in EGF and rs2010963 in VEGF were significantly correlated with the clinical outcome of patients with advanced ESCC (Table 2). Meanwhile, the SNPs in EGFR and PIK3CA also showed a borderline significant effect for prognosis. According to the cumulative effects analysis, patients carrying more unfavorable genotypes exhibited a significant increased risk of poor survival (Table 4 and Fig. 1). Finally, serum EGF and VEGF levels exhibited an obvious association with the prognosis in patients with advanced ESCC (Fig. 2 and Table S2).

The EGF:rs4444903 SNP localized at position 61 (+61A/G) in the 5′-untranslated region of EGF. The G/G genotype of the EGF +61A/G polymorphism has been observed to correlate with a greater risk of esophageal adenocarcinoma and increased levels of EGF in gastroesophageal reflux disease patients compared with A/A or A/G [37]. It has also shown a significant association with survival among ESCC patients treated with radiotherapy [38]. In the current study, we demonstrated that the G/G genotype of rs4444903 was significantly associated with increased risk of adverse clinical outcomes in ESCC patients with advanced tumor stages. We also observed that G/G showed a trend to correlate with elevated serum EGF levels in these patients. Because EGF expression contributed to poor survival in our ESCC population, we suggest that the adverse effect associated with the G/G genotype may partially exert by increased expression of EGF.

EGF:rs2237051 (A/G) is a non-synonymous SNP in the coding region of the EGF gene which results in a change from isoleucine (ATA) to methionine (ATG) at amino acid position 708 (I708M).

The human EGF precursor is a transmembrane protein with 1207 amino acids (aa) (prepro-EGF) which is encoded from a gene containing 24 exons [39]. Mature EGF is encoded by exons 20 and 21 with only 53 amino acids (aa 971-1023) [40]. It is released by its precursor prepro-EGF by cleavage of Arg-Asn and Arg-His bonds [39]. The EGF:rs2237051, I708M, is located within one of eight EGF-like repeat domains of prepro-EGF encoded by exons 6, 7, 8, 9, 15, 17, 18 and 19 [39]. The variant carriers of EGF:rs2237051 have been reported to be associated with a markedly decreased risk of lung cancer [41]. Generally, the possible function of this polymorphic site is not well understood. In our results, we also demonstrated that the variant allele G of EGF:rs2237051 was associated with favorable prognosis in advanced ESCC patients. Prepro-EGF has been demonstrated to stimulate autophosphorylation of the EGF receptor in membrane vesicles from A-431 cells and acts as a growth factor to support the growth of EGF-dependent mouse keratinocytes [42], [43]. Whether or not the genetic variants of EGF:rs2237051 correlated with the function of prepro-EGF needs further investigation.

The association of the SNPs of VEGF at -460T/C (rs833061), 405G/C (rs2010963 or -634G/C), and 936C/T (rs3025039) with clinical outcome of patients with esophageal cancer has been investigated previously [23]. No independent associations were found for VEGF -460T/C and 405 G/C. The combined CGC haplotype of the three VEGF SNPs (-460T/C, 405G/C, and 936C/T) was associated with worse overall survival of esophageal cancer patients [23]. In the current study, we demonstrated that VEGF rs2010963 independently correlated with prognosis of patients with advanced ESCC. The C/C variant is evidently unfavorable for clinical outcome. VEGF rs2010963 405G/C has been found to correlate with VEGF protein production stimulated by lipopolysaccharide [44]. However, we did not find an association between the genotypes of rs2010963 and serum VEGF levels in patients with ESCC (Fig. 2). Because the sera were collected before patients received treatment, we suggest that the genotypes of rs2010963 may be associated with post-treatment expression of VEGF and thereby influence the survival outcome of patients.

Even though EGF:rs4444903, EGF:rs2237051 and VEGF:rs2010963 were each significantly associated with a better prognosis of advanced ESCC, we found the cumulative effect of VEGF:rs2010963 with either EGF:rs4444903 or EGF:rs2010963 to be even better than the cumulative effect of these 3 SNPs combined (Table 4). Joint analysis of EGFR:rs2227983 and the SNPs of the downstream signaling factor PIK3CA, revealed strong cumulative effects for both increased risk of death and for recurrence with increasing numbers of unfavorable genotypes in both EGF:rs2237051 and EGF:rs4444903 sets (Table 4). The minor allele frequencies of the 3 SNPs of PIK3CA, rs6333624, rs7651263 and rs7621329 were similar (1.5%) in our study population. In a previous study, the minor allele homozygous AA of PIK3CA:rs6443624 was associated with risk of recurrence [45], but none of these PIK3CA SNPs were found to correlate with recurrence in esophageal cancer patients [33]. In our study, all the minor alleles of these PIK3CA SNPs show borderline significant adverse effects on survival of advanced ESCC patients. Meanwhile, the minor allele of PIK3CA:rs6443624 had a cumulative effect with the unfavorable genotypes of EGF, VEGF and EGFR.

The role of EGFR:rs2227983 (G to A, R497K) in cancer has been extensively investigated. EGFR with an argnine-to-lysine substitution has been reported to exhibit a significantly reduced growth response to EGF and TGF-α [46]. The homozygous argnine allele has been suggested to associate with favorable prognosis in patients with colorectal carcinoma [47], whereas it has no evident effect on the outcome of esophageal cancer patients [38]. R497K alone does not significantly associate with risks of esophageal cancer and gastric cancer; however, the arginine allele has shown a cumulative effect of increasing hazard of death in combination with other SNPs [18], [48]. We found, unexpectedly, that patients carrying the lysine allele (GA or AA genotypes) showed an increased trend of risk for death and disease recurrence, though without reaching statistical significance (P = 0.058, Table 2). The A allele of EGFR also performed interactively with SNPs in the EGF, VEGF, and PIK3CA genes. The function of R497K in ESCC cells is not clear and merits further investigation.

EGFR expression is frequently detected in esophageal squamous cell carcinoma [49], [50] and adenocarcinoma. Over-expression of EGFR has been associated with poor prognosis of patients with esophageal cancer [51], [52]. Here we have investigated the association of EGFR expression and prognosis in our ESCC study population. In agreement with previous observations, EGFR expression was frequently detected in tumor tissue but not in normal tissue. However, unexpectedly, we did not observe the association of EGFR over-expression and prognosis of ESCC (data not shown). Interestingly, we clearly observed that over-expression of EGF significantly associated with adverse survival outcome in our patients.

Many drugs targeted for cancer have been developed or are under development based on the results of studies focusing on the prognostic relevance of receptor tyrosine kinases and their growth factor ligands. Therapeutic targeting of EGF/EGFR and VEGF/VEGFR signaling is a major approach of anti-cancer therapy. However, unlike non-small cell lung cancer (NSCLC), where useful markers such as EGFR gene mutation have already been established, the molecular markers regulating sensitivity to EGF/EGFR as well as those targeting VEGF/VEGFR in ESCC are hardly known. Based on our results, we propose a therapeutic approach for treating ESCC by considering genetic polymorphisms and the serum level of EGF and VEGF as biomarkers. The main limitations of the study are that it lacks a larger prospective study cohort and a multi-center validation.

In conclusion, hereditary genetic polymorphisms and the expression levels of EGF and VEGF can serve as prognostic predictors of patients with advanced ESCC, and provide insight for optimizing personalized therapy for patients with ESCC.

## Supporting Information

Table S1 **The effects of 26 growth-factor related SNPs on the overall survival of 95 randomly selected advanced ESCC subjects.**
(DOC)

Table S2 **Association of EGF or VEGF expression and the prognosis of patients with advanced ESCC.**
(DOC)
