# Supplementary material for: Genetic Variants of EGF and VEGF Predict Prognosis of Patients with Advanced Esophageal Squamous Cell Carcinoma
Source: PLoS One. 2014 Jun 19;9(6):e100326. doi: 10.1371/journal.pone.0100326 (PMC4063891; doi:10.1371/journal.pone.0100326)
Supplement: Table S1 — The effects of 26 growth-factor related SNPs on the overall survival of 95 randomly selected advanced ESCC subjects. (DOC) [file pone.0100326.s001.doc]

**Table S1.** The effects of 26 growth-factor related SNPs on the overall survival of 95 randomly selected advanced ESCC subjects

|  |  |  |  | **Overall survival** | |
| --- | --- | --- | --- | --- | --- |
| **GENE** | **SNPs** | **Genotypes** | **N** | ***Adjusted HRs (95% CI)** | **p-value** |
| EGFR | rs2017000 (intron) | GG | 31 | 1 |  |
|  |  | GA | 46 | 0.73 (0.45-1.18) | 0.196 |
|  |  | AA | 18 | 0.73 (0.39-1.38) | 0.339 |
| EGFR | rs759165 (intron) | GG | 79 | 1 |  |
|  |  | GA | 16 | 1.02 (0.58-1.80) | 0.947 |
| EGFR | rs3735061 (intron) | CC | 51 | 1 |  |
|  |  | TC | 40 | 0.72 (0.45-1.16) | 0.181 |
|  |  | TT | 4 | 0.97 (0.34-2.76) | 0.948 |
| EGFR | rs2293347(coding-syn ) | GG | 50 | 1 |  |
|  |  | GA | 40 | 0.91 (0.58-1.44) | 0.912 |
|  |  | AA | 5 | 0.86 (0.33-2.23) | 0.862 |
| EGFR | rs1140475 (coding-syn ) | CC | 82 | 1 |  |
|  |  | TC | 12 | 1.38 (0.72-2.66) | 0.331 |
|  |  | TT | 1 | 1.30 (0.17-9.90) | 0.803 |
| EGFR | rs6958497 (intron) | TT | 78 | 1 |  |
|  |  | CT | 15 | 0.73 (0.37-1.45) | 0.370 |
|  |  | CC | 2 | 0.63 (0.15-2.74) | 0.541 |
| EGFR | rs2227983 (missense) | GG | 27 | 1 |  |
|  |  | GA | 36 | 1.61 (0.95-2.74) | 0.080 |
|  |  | AA | 32 | 1.79 (1.03-3.13) | **0.041** |
| EGFR | rs35515689 | AA | 95 | - | - |
|  |  |  |  |  |  |
| EGF | rs4444903 (5’UTR) | AA | 40 | 1 |  |
|  |  | GA | 1 | 0.25 (0.03-2.02) | 0.193 |
|  |  | GG | 54 | 1.68 (1.07-2.63) | **0.024** |
| EGF | rs2237051 (missense) | AA | 48 | 1 |  |
|  |  | GA | 38 | 0.68 (0.43-1.08) | 0.103 |
|  |  | GG | 9 | 1.29 (0.61-2.74) | 0.510 |
| IGF1R | rs2272037 (intron) | GG | 60 | 1 |  |
|  |  | GA | 26 | 1.92 (1.17-3.14) | **0.010** |
|  |  | AA | 9 | 0.82 (0.38-1.78) | 0.622 |
| IGF1R | rs2229765 (coding-syn ) | GG | 36 | 1 |  |
|  |  | GA | 44 | 0.79 (0.47-1.35) | 0.388 |
|  |  | AA | 15 | 0.98 (0.51-1.89) | 0.956 |
| IGF1R | rs2016347 (3’UTR) | AA | 29 | 1 |  |
|  |  | CA | 47 | 1.37 (0.83-2.25) | 0.222 |
|  |  | CC | 19 | 1.70 (0.86-3.35) | 0.126 |
| IGF1 | rs7136446 (intron) | TT | 64 | 1 |  |
|  |  | CT | 27 | 0.88 (0.53-1.44) | 0.603 |
|  |  | CC | 4 | 9.86 (2.99-32.49) | **<0.001** |
| IGF1 | rs2946834 (3’UTR) | CC | 30 | 1 |  |
|  |  | TC | 33 | 1.03 (0.60-1.74) | 0.927 |
|  |  | TT | 32 | 1.19 (0.69-2.05) | 0.543 |
| VEGF | rs3025040 (3’UTR) | CC | 55 | 1 |  |
|  |  | TC | 36 | 0.73 (0.46-1.17) | 0.193 |
|  |  | TT | 4 | 0.88 (0.30-2.56) | 0.807 |
| VEGF | rs25648 (coding-syn ) | CC | 76 | 1 |  |
|  |  | CT | 19 | 0.73 (0.42-1.27) | 0.264 |
| VEGF | rs2010963 (5’UTR) | GG | 35 | 1 |  |
|  |  | CG | 47 | 0.94 (0.59-1.49) | 0.790 |
|  |  | CC | 13 | 2.51 (1.25-5.05) | **0.010** |
| VEGF | rs3025039 (3’UTR) | CC | 57 | 1 |  |
|  |  | CT | 34 | 0.69 (0.42-1.11) | 0.121 |
|  |  | TT | 4 | 0.85 (0.29-2.47) | 0.761 |

|  |  |  |  | **Overall survival** | |
| --- | --- | --- | --- | --- | --- |
| **Gene** | **SNPs** | **Genotypes** | **N** | **Adjusted HRs (95% CI)** | **p-value** |
| PIK3CA | rs6443624 (intron) | CC | 78 | 1 |  |
|  |  | CA | 16 | 2.09 (1.15-3.80) | **0.016** |
|  |  | AA | 1 | 0.50 (0.07-3.76) | 0.498 |
| PIK3CA | rs7651265 (intron) | AA | 79 | 1 |  |
|  |  | AG | 15 | 1.94 (1.06-3.58) | **0.032** |
|  |  | GG | 1 | 0.49 (0.07-3.70) | 0.489 |
| PIK3CA | rs7621329 (intron) | CC | 78 | 1 |  |
|  |  | TC | 16 | 2.09 (1.15-3.80) | **0.016** |
|  |  | TT | 1 | 0.50 (0.07-3.76) | 0.498 |
| AKT1 | rs1130214 (5’UTR) | GG | 75 | 1 |  |
|  |  | GT | 15 | 1.04 (0.57-1.88) | 0.906 |
|  |  | TT | 5 | 4.62 (1.64-13.00) | **0.004** |
| AKT2 | rs892119 (intron) | GG | 73 | 1 |  |
|  |  | GA | 22 | 1.52 (0.92-2.53) | 0.104 |
| FRAP1 | rs11121704 (intron) | TT | 85 | 1 |  |
|  |  | TC | 10 | 2.74 (1.33-5.64) | **0.006** |
| PTEN | rs2299939 (intron) | CC | 61 | 1 |  |
|  |  | CA | 32 | 1.10 (0.69-1.75) | 0.681 |
|  |  | AA | 2 | 0.68 (0.16-2.99) | 0.612 |

***Adjusted for stage, age, CCRT, and OP**
