# Supplementary material for: Genetic Variants of EGF and VEGF Predict Prognosis of Patients with Advanced Esophageal Squamous Cell Carcinoma
Source: PLoS One. 2014 Jun 19;9(6):e100326. doi: 10.1371/journal.pone.0100326 (PMC4063891; doi:10.1371/journal.pone.0100326)
Supplement: Table S2 — Association of EGF or VEGF expression and the prognosis of patients with advanced ESCC. (DOC) [file pone.0100326.s002.doc]

**Table S2**. Association of EGF or VEGF expression and the prognosis of patients with advanced ESCC

|  |  | **ESCC patients with advanced stages (N=105)** | | | | |  |
| --- | --- | --- | --- | --- | --- | --- | --- |
|  |  | **Overall survival** | |  | **Progression-free survival** | | |
| **Expression** | **N** | ***Adjusted HRs**  **(95% CI)** | **p-value** | ***Adjusted HRs**  **(95% CI)** | | **p-value** |  |
| **EGF** |  |  |  |  | |  |  |
| Undetectable | 33 | 1 |  | 1 | |  |  |
| Detectable | 82 | 1.72 (0.93-3.18) | 0.083 | 0.94 (0.58-1.51) | | 0.794 |  |
| **VEGF** |  |  |  |  | |  |  |
| Undetectable | 69 | 1 |  | 1 | |  |  |
| Detectable | 46 | 1.89 (1.22-2.92) | **0.005** | 1.57 (1.04-2.38) | | **0.032** |  |
|  |  |  |  |  | |  |  |

* Adjusted for stages, surgical status and CCRT
